# Maxson’s Practice of Medicine

**Published:** 1861-09

**Authors:** 


### MAXSON'S PRACTICE OF MEDICINE.

A Treatise on the Practice of Medicine. By Edwin R. Maxon, M. D., formerly Lecturer on the Institutes and Practice of Medicine in the Geneva Medical College. Philadelphia: Lindsay and Blackiston, 1861, 705 pages.

We have received from the author a copy of the above work. It comes to us in the very best style of Messrs. Lindsay & Blackiston. It claims to be the result of an extended practice and careful observation, with such aid as could be gleaned from the writings of various authors of merited celebrity. It seems to be in fact mainly the expression of the author's individual opinions. We make the following quotations from the work as defending this course, and acknowledging this view: "While I have preferred giving my own opinion of remedies, and in fact almost every thing else of which I have treated, it has been from no feeling of ostentation, but simply because I believe I am better qualified to give my own opinion, than to uphold the opinions of other men, which in turn they can do much better than I can do it for them, but this certainly can detract nothing from the value of the work, as I am indebted to the *good*, and *wise*, and *great*, of the Medical Profession, as well as to my own observation, for the opinions which I hold in relation to the principles, science and practice of medicine."

The author has included in his work most or all the diseases usually treated in works upon Theory and Practice of Medicine, and some usually regarded as Surgical diseases. As showing the general plan of the work we make a quotation from the preface: "It will be seen that I have glanced at the Anatomy and Physiology, as I have taken up the diseases peculiar to each part of the human system. This I have done in part to make the work more valuable to those practitioners of medicine who have not time to review Anatomy and Physiology; but more especially to keep the mind of the reader fixed upon the diseased part, and its conditions; thus rendering the work not only more valuable, but I trust more interesting."

